# Exploring the Relationship Between Serum Neuronal Pentraxin 2 and Poststroke Cognitive Impairment in Patients With First‐Episode Acute Ischemic Stroke

**DOI:** 10.1002/brb3.70305

**Published:** 2025-02-09

**Authors:** Jie Li, Wenyang Ma, Shiyuan Gu

**Affiliations:** ^1^ Department of Neurology The Affiliated Yixing Hospital of Jiangsu University Yixing China; ^2^ Department of Neurology The Affiliated Yixing Clinical School of Medical School of Yangzhou University Yixing China

**Keywords:** acute ischemic stroke, Montreal Cognitive Assessment, neuronal pentraxin 2, poststroke cognitive impairment

## Abstract

**Background and Objective**: Neuronal pentraxin 2 (NPTX2) is associated with cognitive impairment in some neurodegenerative diseases. However, few studies focused on the association between NPTX2 and poststroke cognitive impairment (PSCI). Hence, this study aimed to investigate the association between serum NPTX2 levels and PSCI.

**Methods**: A total of 134 participants with acute ischemic stroke (AIS) and 42 normal controls were enrolled in this study. Admission baseline information was collected, and serum NPTX2 levels were determined within 24 h using enzyme‐linked immunosorbent assay (ELISA) at hospital admission. All subjects were evaluated for cognitive function using the MoCA (Montreal Cognitive Assessment) scale at 3 months after stroke onset, and patients with AIS were divided into PSCI and PSNCI (poststroke no cognitive impairment) groups, with a total MoCA score < 26 defined as PSCI. This study analyzed the relationship between serum NPTX2 and MoCA score and the risk factors of PSCI. The receiver operating characteristic (ROC) curve was to evaluate the diagnostic value of serum NPTX2 levels on PSCI.

**Results**: Among the 134 AIS participants, 53 (38.8%) patients suffered from PSCI at 3 months after stroke onset. The serum levels of NPTX2 in the PSCI group, PSNCI group, and normal controls group were significantly different (*p* < 0.05). The serum NPTX2 levels in the PSCI and PSNCI groups were higher than normal control group, and the serum NPTX2 levels in the PSCI group were lower than PSNCI group (*p* < 0.05). Serum NPTX2 levels were positively correlated with the total score of MoCA (*r* = 0.329, *p* < 0.01), and also positively correlated with some subcognitive domains of MoCA (visuospatial and executive functions, naming, delayed memory, and attention). ROC curve indicated that serum NPTX2 predicted cognitive impairment in AIS patients. Multivariate Logistic regression analysis indicated serum NPTX2 was an independent protective factor for PSCI (odds ratio [OR] = 0.075, 95% CI 0.010–0.812, *p* < 0.01).

**Conclusions**: Lower serum NPTX2 levels were associated with PSCI within 3 months in patients with first‐episode AIS. Lower levels of serum NPTX2 may be associated with impairment in visuospatial and executive functions, naming, delayed memory, and attention, while a further larger‐scale study is needed to verify our findings.

## Graphical abstract

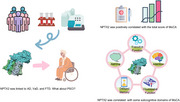

## 1 Introduction

Stroke is the second leading cause of death and the third leading cause of disability worldwide (GBD 2019 Stroke Collaborators 2021). Stroke increases the risk of cognitive impairment, which affects up to 80% of stroke survivors (Sun, Tan, and Yu 2014; Qu et al. 2015). In addition, several studies have shown that about one‐third of patients suffered from cognitive impairment at 3 months after first‐episode stroke onset (Boutros et al. 2023; Obaid et al. 2020). Poststroke cognitive impairment (PSCI) is a clinical syndrome of cognitive dysfunction after stroke and can be easily overlooked (Kim, Shin, and Chang 2022). PSCI increases the risk of death in stroke survivors, leads to worsen prognostic function outcomes in patients with acute ischemic stroke (AIS) (Jokinen et al. 2015), and severely affects the daily life of AIS patients (Irfani Fitri, Fithrie, and Rambe 2020). Therefore, PSCI hinders the rehabilitation of stroke survivors, posing challenges for their reintegration into society and placing a greater burden on both families and society. Early identification of the cognitive functional status of AIS patients and active interventions are expected to reduce the risk of poor stroke prognosis and reduce the socioeconomic burden. Previous studies have shown that blood molecular biomarkers can be used for the early prediction of PSCI (Kim, Shin, and Chang 2022). Nevertheless, there are no precise and reliable molecular markers that can be turned into clinical applications.

Neuronal pentraxin 2 (NPTX2) is a member of the pentraxins family which contains a signal peptide sequence and three N‐glycosylation sites (Chapman, Shanmugalingam, and Smith 2019). NPTX2 aggregates with Neuronal pentraxin 1 (NPTX1) and Neuronal pentraxin receptor (NPTXR) to form multimeric complexes that promote synaptogenesis and synaptic remodeling in cortical regions and hippocampal pyramidal neurons (Sia et al. 2007). These complexes, along with α‐amino‐3‐hydroxy‐5‐methyl‐4‐isoxazole propionic acid receptor (AMPAR), co‐localize on the cell membrane surface and bind to AMPA selective glutamate receptor subunits, promoting glutamatergic excitatory synaptic transmission (O'Brien et al. 1999). NPTX2 is pivotal in synapse formation, synaptic remodeling, and excitatory synaptic transmission (Sia et al. 2007; O'Brien et al. 1999). In recent years, studies on the cognitive function of NPTX2 in neurodegenerative diseases have been increasing. One previous study has found that cerebrospinal fluid (CSF) NPTX2 levels are closely related to cognitive function in patients with Alzheimer's disease (AD) (Galasko et al. 2019). In addition, the level of NPTX2 expression was closely associated with cognitive function in vascular dementia (Shao et al. 2020) and frontotemporal dementia (van der Ende et al. 2020), and negatively correlated with the severity of disease.

The pathophysiological mechanisms of PSCI are unclear yet (Sun, Tan, and Yu 2014). Previous studies have shown that stroke causes impairment of synaptic plasticity, which may cause PSCI (Joy and Carmichael 2021). Therefore, it is necessary to investigate and analyze the relationship between serum NPTX2 and poststroke cognitive impairment in patients with AIS.

## 2 Materials and Methods

### 2.1 Study Population

**FIGURE 1:**
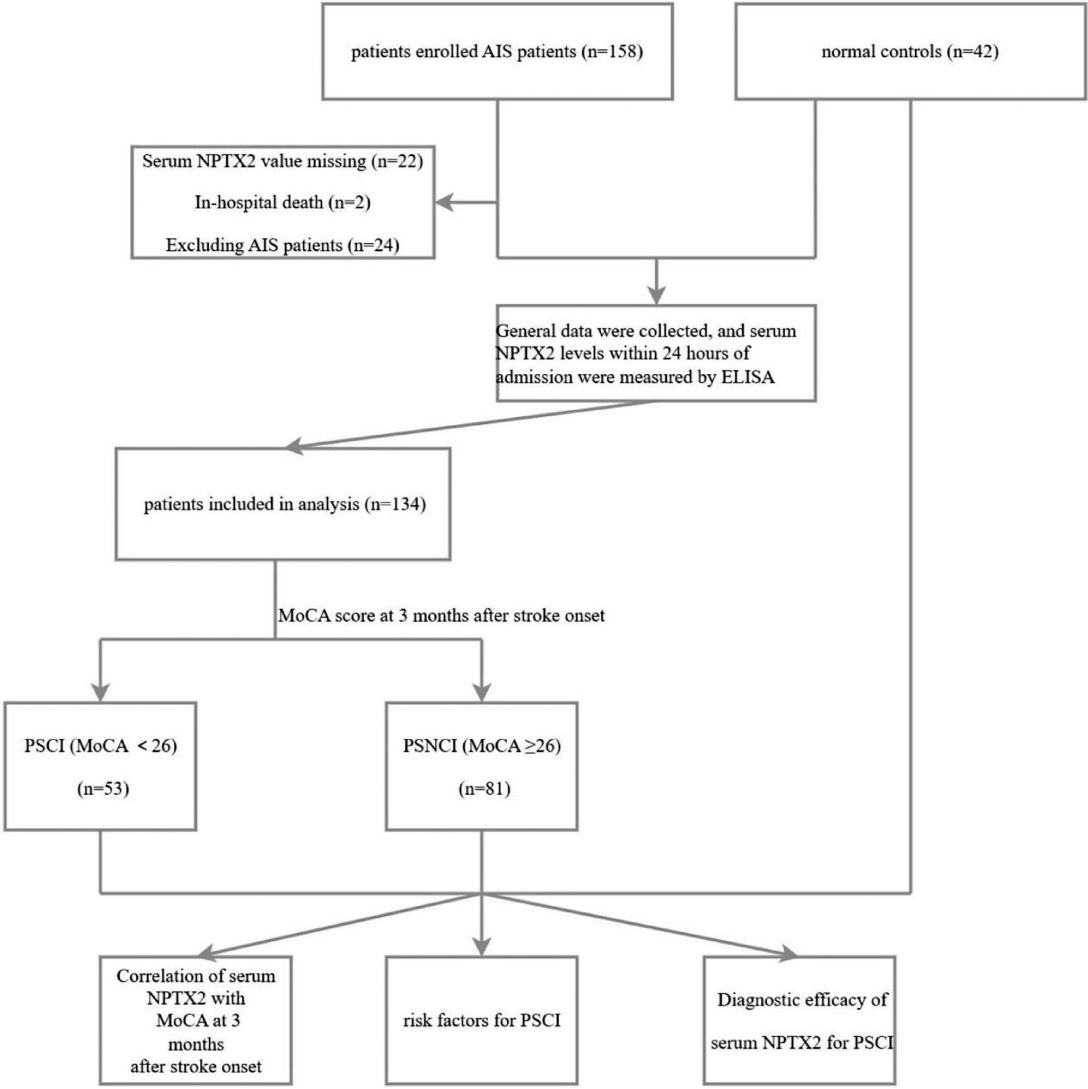
Flow chart of this study.

This study is a prospective cohort study. We recruited patients with first‐episode AIS and collected their baseline data upon admission. Cognitive function was assessed 3 months later to evaluate the occurrence of PSCI. We prospectively recruited the first onset patients with AIS and normal controls from June 2022 to November 2022 in Yixing People's Hospital. The inclusion criteria for patients in this study were as follows: (1) diagnosis with AIS based on clinical history, neurological symptoms, and brain CT scans (Hasan et al. 2018); (2) diagnosed and treated within 72 h of the symptom onset (limiting the inclusion to patients within 72 h helps minimize variability in NPTX2 levels that could arise from delayed sampling, thus providing a more reliable assessment); and (3) subjects or delegated family members voluntarily and sign the informed consent form for this trial. The exclusion criteria were as follows: (1) past history of cognitive dysfunction; (2) past history of cerebral hemorrhage, tumors, severely infectious or autoimmune diseases; (3) preexisting neurological disorders that interfere with neurological assessment, such as Alzheimer's disease (AD) or Parkinson's disease (PD); (4) those whose condition changed after enrollment and did not meet the inclusion criteria; and (5) patients with incomplete information recording or incomplete data collection. Furthermore, normal controls were enrolled from contemporaneous physical examination centers or volunteers with gender, age, and education matched to the case group, with complete data, without prior history of stroke, cognitive impairment or dementia. Due to practical limitations in volunteer recruitment, the control group was slightly smaller than the case group. The sample size calculation was primarily based on the case group, as the study's main objective was to explore the relationship between NPTX2 levels and PSCI in stroke patients. The control group mainly served to provide reference values for NPTX2 levels. Two patients with AIS suffered from in‐hospital death and serum NPTX2 levels were not assayed in 22 AIS patients. Eventually, a total of 134 AIS patients and 42 normal controls were enrolled in this study (Figure 1).

### 2.2 Standard Protocol Approvals and Patient Consents

This study was performed following Helsinki Declaration and was approved by the Ethical Committee of Yixing People's Hospital. Written informed consent was signed by all participants or their legal representatives before this study began.

### 2.3 Baseline Data Collection

Upon admission, all participants were comprehensively and precisely underwent a detailed medical history, physical examination, and several necessary tests. A neurologist collected all of the baseline characteristics including demographic information, medical history, vascular risk factors, and important laboratory data. General information included age, sex, education levels, body mass index (BMI), hypertension, diabetes mellitus, hyperlipidemia, coronary artery disease (CHD), atrial fibrillation, regular smoking, heavy drinking, and blood pressure levels. Important laboratory parameters consisted of hypersensitive C‐reactive protein (hs‐CRP), white blood cell (WBC) counts, hemoglobin (Hb), fasting blood glucose (FBG), glycated hemoglobin (HbA1C), uric acid (UA), homocysteine (HCY), total cholesterol (TC), triglyceride (TG), high‐density lipoprotein cholesterol (HDL‐C), and Low‐density lipoprotein cholesterol (LDL‐C) levels. In addition, the mRS (modified Rankin Scale) score at discharge, and National Institute of Health Stroke Scale (NIHSS) score on admission, were assessed. Etiological classification of AIS was based on the Trial of Org 10172 in Acute Stroke Treatment (TOAST) type criteria (Adams et al. 1993).

### 2.4 Blood Sampling and Determination of NPTX2 Levels

Fasting blood samples were collected in EDTA anticoagulation blood collection within 24 h after admission. Specimens were refrigerated at 4°C for 30 min, centrifuged at 3000 r/min for 15 min at the central laboratory. The isolated serum was then dispensed into labeled enzyme‐free EP tubes and stored at –80°C for later testing. The serum concentrations of NPTX2 were determined using an enzyme‐linked immunosorbent assay (ELISA) reagent (Novus Biologicals, Minneapolis, MN, USA). All experimental protocols referred to reagent instructions and serum NPTX2 levels were measured in ng/mL.

### 2.5 Follow‐Up and Outcome Evaluation

All studied patients with AIS received conventional treatments such as antiplatelet or anticoagulation, lipid‐lowering, vasodilators, reduction of cerebral edema, controlling blood pressure and glucose levels, but without medication to improve cognitive function. The Montreal Cognitive Assessment (MoCA) scale was superior to the Mini‐Mental State Examination (MMSE) scale for cerebrovascular diseases, detecting significantly more cognitive impairment than the MMSE (Pendlebury et al. 2010). Cognitive functions were assessed by a trained neurologist using the MoCA scale, and 3 months after stroke onset. The MoCA scale with a total score of 30 points covers subcognitive domains of visuospatial and executive function, naming, delayed memory, attention, language, abstraction, and orientation. Subjects with less than 12 years of education add 1 point to the total score. The outcome of this study was divided into the poststroke no cognitive impairment (PSNCI) group and the PSCI group, and PSCI was defined as a total MoCA score of less than 26 (Pendlebury et al. 2010).

### 2.6 Statistical Analyses

The data analyses were conducted using the Statistical Package for Social Science (SPSS, version 25.0), and graphs were generated using the software GraphPad Prism Version 8.0. Categorical variables are expressed as numbers (proportions) and were compared using the chi‐square test or Fisher's exact tests. Continuous variables are presented as means (standard deviation, SD) or median (interquartile range, IQR) and were analyzed by using *t*‐test, Mann–Whitney *u*‐test, analysis of variance, or Kruskal–Wallis *H*‐test depending on the specific case. Bonferroni corrections were applied to each test to adjust for multiple testing when the Kruskal–Wallis H test, chi‐square test, or Fisher's exact tests showed significant differences between the groups. Following the analysis of variance for the three groups, the LSD test was applied to each test to adjust for multiple testing. Thereafter, spearman's rank correlation test was used to analyze the correlation between admission serum NPTX2 expression levels and a total MoCA score. The receiver operating characteristic (ROC) curve was plotted to evaluate the diagnostic value of serum NPTX2 on PSCI. Multivariate Logistic regression analysis was performed to detect the risk factors for PSCI. In the multivariate logistic regression analysis, based on the cut‐off value of NPTX2 derived from the ROC curve (0.986 ng/mL), we classified NPTX2 into two categories, high NPTX2 (≥0.986 ng/mL) and low NPTX2 (< 0.986 ng/mL), and used it as a binary categorical variable for analysis. All analyses were two‐sided, and *p* < 0.05 was considered statistically significant.

## 3 Results

### 3.1 Baseline Characteristics of This Study Population

Among the 134 participants in this study, 53 (38.8%) patients suffered from PSCI at the 3‐month follow‐up after stroke onset, of whom 33 (62.3%) individuals were male and 20 (37.7%) patients were female. The characteristics of the PSCI, PSNCI patients, and normal control participants are shown in Table 1. We found a significant difference in serum NPTX2 between PSCI group and PSNCI group (0.83 [0.67–0.91] ng/mL vs. 0.91 [0.73–1.11] ng/mL, *p* < 0.05). Additionally, elevated levels of serum NPTX2 were found between PSCI group and normal controls (0.83 [0.67–0.91] ng/mL vs. 0.47 [0.44–0.49] ng/mL, *p* < 0.05). Compared with the PSNCI group, patients in the PSCI group had a higher proportion of dyslipidemia, higher admission NIHSS score, and higher mRS score at discharge (*p* < 0.05). There were no significant differences in the other confounders, such as age, sex, BMI, education, other vascular risk factors, SBP, DBP, TOAST classification, WBC counts, hemoglobin, glucose, HbA1C, hs‐CRP, uric acid, homocysteine, Cholesterol, Triglyceride, LDL‐C, and HDL‐C.

**TABLE 1:** Baseline characteristics of study samples.

Variables	PSCI patients (*n* = 53)	PSNCI patients (*n* = 81)	Normal controls (*n* = 42)	*F*/*H*/$\chi^{2}$	*p‐*value
Age, mean (SD), years	68.60 ± 10.47	65.93 ± 12.51	64.45 ± 10.85	1.633^a^	0.19
Sex, *n* (%)				4.916^b^	0.08
Male	33 (62.3%)	61 (75.3%)	24 (57.1%)		
Female	20 (37.7%)	20 (24.7%)	18 (42.9%)		
BMI, mean (SD), kg/m^2^	24.15 ± 3.18	24.74 ± 3.60	24.63 ± 3.26	0.511^a^	0.60
Education, *n* (%)				2.563^b^	0.28
≤12 years	45 (84.9%)	64 (79.0%)	30 (71.4%)		
> 12 years	8 (15.1%)	17 (21.0%)	12 (28.6%)		
Medical history, *n* (%)					
Hypertension	48 (90.6%)	63 (77.8%)	25 (59.5%)^*^	12.879^b^	< 0.01
Diabetes mellitus	31 (58.5%)	33 (40.7%)	12 (28.6%)^*^	8.913^b^	0.01
Dyslipidemia	34 (64.2%)	33 (40.7%)^*^	18 (64.2%)	7.685^b^	0.02
CHD	7 (13.2%)	9 (11.1%)	3 (7.1%)	0.910^b^	0.63
Atrial fibrillation	5 (9.4%)	7 (8.6%)	1 (2.4%)	2.050^b^	0.36
History of smoking	29 (54.7%)	38 (46.9%)	6(14.3%)^*^, ^┼^	17.607^b^	< 0.01
History of drinking	7 (13.2%)	21 (25.9%)	2(4.8%)^┼^	9.551^b^	< 0.01
NIHSS score on admission, median (IQR)	4 (3–6)	3 (1–4)^*^		−4.550^c^	< 0.01
mRS score at discharge, median (IQR)	3 (2–4)	2 (1–2)^*^		−6.504^c^	< 0.01
SBP, mean (SD), mmHg	158.43 ± 21.19	24.74 ± 3.60	137.50 ± 14.36	1.511^a^	0.22
DBP, mean (SD), mmHg	88.47 ± 11.61	88.14 ± 11.40	85.02 ± 10.38	1.349^a^	0.26
TOAST classification, *n* (%)				0.747^b^	0.86
Large artery atherosclerosis	39 (73.6%)	57 (70.4%)			
Cardioembolism	3 (5.7%)	5 (6.2%)			
Small vessel disease	11 (20.8%)	18 (22.2%)			
Other/unknown	0 (0.0%)	1 (1.2%)			
Laboratory tests					
WBC counts, mean (SD), ×10^9^/L	7.13 ± 2.52	6.64 ± 2.00	5.98 ± 2.11^*^	3.236^a^	0.04
Hemoglobin, mean (SD), g/L	137.34 ± 18.34	142.07 ± 20.64	137.33 ± 17.82	1.314^a^	0.27
Glucose, mean (SD), mmol/L	6.55 ± 2.17	6.41 ± 2.37	5.45 ± 1.39^*^, ^┼^	3.785^a^	0.03
HbA1C, median (IQR), %	6.20 (5.60–7.25)	6.00 (5.50–7.50)	5.70 (5.40–6.42)	4.670^c^	0.09
hs‐CRP, median (IQR), mg/L	2.11 (0.98–3.88)	1.48 (0.71–3.04)	1.04 (0.54–2.25)	6.603^c^	0.04
Uric acid (µmol/L)	307.96 ± 99.40	332.23 ± 96.17	322.18 ± 70.05	1.122^a^	0.33
Homocysteine, median (IQR), µmol/L	13.70 (11.30–16.80)	14.40 (11.80–17.65)	11.70 (10.42–14.18)^┼^	10.463^c^	< 0.01
Cholesterol, mean (SD), mmol/L	4.86 ± 1.45	4.61 ± 1.23	4.78 ± 0.89	0.837^a^	0.47
Triglyceride, mean (SD), mmol/L	1.56 ± 0.89	1.67 ± 1.03	1.55 ± 0.67	0.297^a^	0.74
LDL‐C, mean (SD), mmol/L	3.21 ± 1.14	3.03 ± 0.94	3.07 ± 0.69	0.591^a^	0.56
HDL‐C, mean (SD), mmol/L	1.38 ± 0.30	1.30 ± 0.30	1.40 ± 0.24	2.327^a^	0.10
NPTX2, median (IQR), ng/mL	0.83 (0.67–0.91)	0.91(0.73–1.11)^*^	0.47 (0.44–0.49)^*^,^┼^	98.715^c^	< 0.01

*Note*: Data are expressed as mean ± standard deviation or *n* (%) or median (IQR).

^a^ Mean ± SD, ANOVA.

^b^ *n* (%), chi‐square test.

^c^ Median (IQR), Kruskal–Wallis *H* test.

* *p*< 0.05 compared with PSCI.

^┼^ *p*< 0.05 compared with PSNCI.

Abbreviations: SD, standard deviation; BMI, body mass index; CHD, coronary heart disease; DBP, diastolic blood pressure; HDL‐C, high‐density lipoprotein cholesterol; IQR, interquartile range; LDL‐C, hs‐CRP, high‐sensitivity C‐reactive protein; LDL‐C, low‐density lipoprotein cholesterol; mRS, modified Rankin Scale; NIHSS, National Institutes of Health Stroke Scale; NPTX2, neuronal pentraxin 2; SBP, systolic blood pressure; TOAST, Trial of Org 10172 in Acute Stroke Treatment; WBC, white blood cell.

### 3.2 Associations Between Serum NPTX2 Levels and PSCI

**FIGURE 2:**
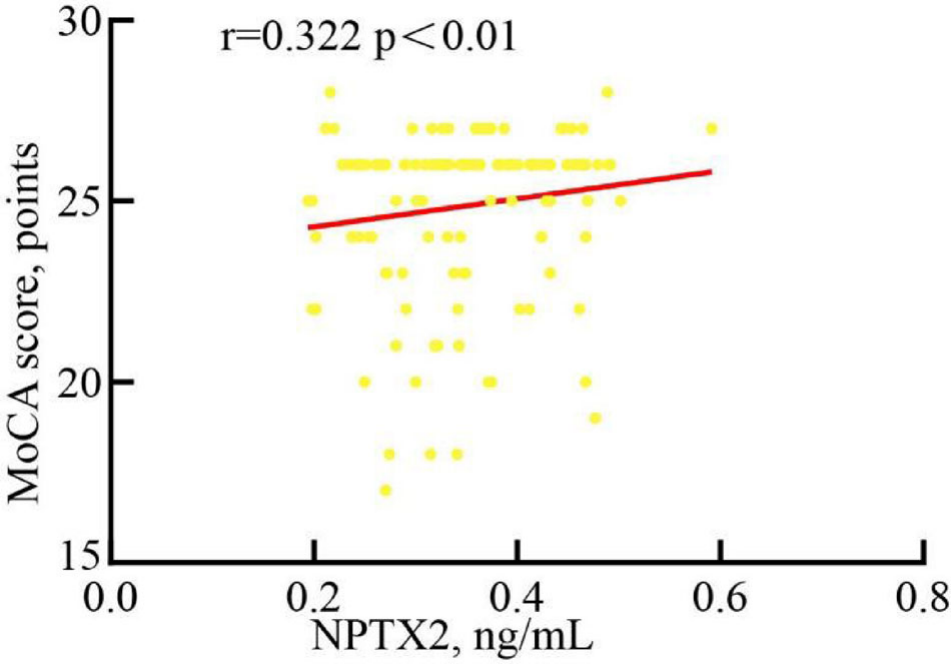
Correlation between serum NPTX2 and 3‐month MoCA total score.

The correlation between NPTX2 and PSCI is presented in Tables 2 and 3 and Figure 2. Firstly, Table 2 showed a significant difference in total MoCA score at 3‐month follow‐up between PSCI and PSNCI groups (22.70 ± 2.25 vs. 26.78 ± 0.51, *p* < 0.01). Additionally, compared to normal controls, patients with PSCI and PSNCI groups had lower MoCA scores, (*p* < 0.05). Furthermore, the correlation between serum NPTX2 and 3‐month total MoCA scores after stroke is shown in Figure 2 and Table 3. Table 3 also showed the relationship of serum NPTX2 with subcognitive domains of MoCA. Serum NPTX2 levels in AIS patients were positively correlated with 3‐month total MoCA scores after stroke (*r* = 0.322, *p* < 0.01). And analyzing the subcognitive domains of MoCA, serum NPTX2 levels were positively related to visuospatial and executive functions (*r* = 0.259, *p* < 0.01), naming (*r* = 0.178, *p* = 0.04), delayed memory (*r* = 0.268, *p* < 0.01), and attention (*r* = 0.238, *p* < 0.01). There was no correlation with language (*r* = 0.131, *p* = 0.13), abstraction (*r* = –0.009, *p* = 0.92), and orientation (*r* = 0.007, *p* = 0.93).

**TABLE 2:** Comparison of total MoCA scores among the three groups at 3 months of follow‐up.

Variable	PSCI patients (*n* = 53)	PSNCI patients (*n* = 81)	Normal controls (*n* = 42)	*F* value	*p‐*value
MoCA score at 3‐month, mean (SD)	22.70 ± 2.25	26.78 ± 0.51^*^	27.02 ± 0.84^*^, ^┼^	155.194	< 0.01

*Note*: Data are expressed as median (IQR).

* *p*< 0.05 compared with PSCI.

^┼^ *p*< 0.05 compared with PSNCI.

Abbreviations: IQR, interquartile range; SD, standard deviation; MoCA, Montreal Cognitive Assessment.

**TABLE 3:** Correlation between admission serum NPTX2 level and total score and subcognitive domains of MoCA at 3 months of stroke onset.

Items	*r* value	*p‐*value
Total score	0.322	< 0.01
Visuospatial and executive functions	0.259	< 0.01
Naming	0.178	0.04
Delayed memory	0.268	< 0.01
Attention	0.238	< 0.01
Language	0.131	0.13
Abstraction	−0.009	0.92
Orientation	0.007	0.93

### 3.3 Diagnostic Value of Serum NPTX2 Levels on PSCI

**FIGURE 3:**
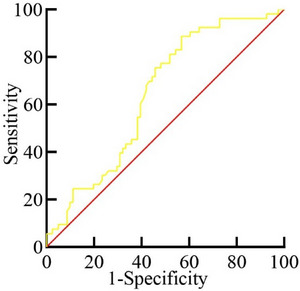
ROC curve analysis of serum NPTX2 levels on the occurrence of PSCI in patients with first‐episode AIS.

The optimal cutoff value for auxiliary diagnosis of PSCI was as follows: serum NPTX2 level < 0.986 ng/mL (sensitivity 88.68%, specificity 43.21%, AUC 0.643, 95% CI 0.550–0.737, *p* < 0.01) (Table 4 and Figure 3).

**TABLE 4:** Diagnostic efficacy of serum NPTX2 levels on PSCI in patients with first‐episode AIS.

variable	AUC	95% confidence interval	Sensitivity (%)	Specificity (%)	*p‐*value	Cut off value (ng/mL)
NPTX2	0.643	0.550–0.737	88.68%	43.21%	< 0.01	< 0.986

### 3.4 Factors Associated With PSCI at 3 Months in First‐Ever AIS Patients

The PSCI and PSNCI groups also have significant differences in dyslipidemia, NIHSS score on admission, mRS score at discharge, and serum NPTX2 (*p* < 0.05; Table 1). The current study further investigated the potential risk factors for PSCI. The results of the multivariate logistic regression analysis, using the NPTX2 cut‐off value (0.986 ng/mL) as a binary categorical variable, showed that lower NPTX2 levels (<0.986 ng/mL) remained an independent risk factor for PSCI (OR = 0.075, 95% CI 0.010–0.812, *p* < 0.01). This result clarifies the association between NPTX2 levels and the risk of PSCI (Table 5).

**TABLE 5:** Multivariate Logistic regression analysis to identify independent factors for the occurrence of PSCI in patients with AIS.

Variable	OR	95% confidence interval	*p‐*value
Dyslipidemia	0.618	0.253–1.506	0.29
NIHSS score on admission	1.150	0.915–1.446	0.23
mRS score at discharge	3.065	1.695–5.543	< 0.01
NPTX2	0.075	0.010–0.812	< 0.01

## 4 Discussion

In this study, we found that baseline serum NPTX2 levels in patients with AIS are significantly associated with PSCI. Specifically, lower serum NPTX2 levels were significantly correlated with PSCI, and multivariable logistic regression analysis identified NPTX2 as an independent protective factor against PSCI. These results suggest that NPTX2 may play a crucial role in maintaining cognitive function following AIS.

The incidence of PSCI was consistent with previous studies, with 53 out of 134 first‐episode AIS patients (38.8%) diagnosed with PSCI 3 months after disease onset, which was consistent with the results of previous studies (Pu et al. 2022; Mohamed Fuad et al. 2020; He et al. 2018; Huang et al. 2022). Serum NPTX2, a novel biomarker of synaptic dysfunction associated with cognitive function in neurodegenerative diseases (Galasko et al. 2019; Shao et al. 2020; van der Ende et al. 2020; Xiao et al. 2017; Swanson and Willette 2016; Nilsson et al. 2023), was positively correlated with total MoCA scores and cognitive subdomains.

The likely explanations for the potential mechanisms between NPTX2 and PSCI are as following: firstly, NPTX2, along with NPTX1 and NPTXR, forms complexes promoting synaptogenesis and remodeling of pyramidal neurons in cortical regions and the hippocampus (Sia et al. 2007), suggesting a correlation between serum NPTX2 and cognitive function in AIS patients. Secondly, lower serum NPTX2 in AIS may impact memory and cognition related to the perirhinal matrix network, influencing synaptic plasticity and brain regulation (Logsdon et al. 2022; Van't Spijker et al. 2019). Thirdly, NPTX2 acts as the AMPA receptor aggregation factor, mediating glutamatergic synaptic transmission. Lower NPTX2 levels may render AIS patients insufficient to counteract excitotoxicity, leading to increased neuronal damage and weaker synaptic plasticity, contributing to PSCI (Schwarz et al. 2002). Therefore, the present study suggests that patients with first‐episode AIS with low levels of serum NPTX2 are more likely to undergo PSCI, and the specific mechanism still needs to be further explored.

Moreover, this study evaluated the diagnostic efficacy analysis of NPTX2 for PSCI. ROC curve analysis was performed to assess the diagnostic accuracy of serum NPTX2 for PSCI at 3 months post‐stroke. The area under the ROC curve (AUC) for serum NPTX2 was 0.643 (*p* < 0.05), indicating moderate diagnostic value. Our results indicate that serum NPTX2 has a moderate diagnostic value for PSCI with high sensitivity, and the diagnostic value of NPTX2 can be further validated and analyzed by increasing the sample size in the future.

Further analysis revealed that NPTX2 levels closely linked to specific cognitive domains. Specifically, the correlation between serum NPTX2 levels and visuospatial and executive functions was the most significant (*r* = 0.259, *p* < 0.01), which may be related to synaptic plasticity disorders in the cortex and hippocampal regions caused by AIS. NPTX2, by forming complexes with NPTX1 and NPTXR, promotes synapse formation and remodeling in these regions, thereby playing a key role in maintaining cognitive function (Sia et al. 2007). Therefore, lower NPTX2 levels may lead to reduced synaptic remodeling capacity, increasing the risk of PSCI. Moreover, the correlation between serum NPTX2 levels and delayed memory (*r* = 0.268, *p* < 0.01) also suggests its importance in memory maintenance. AIS may affect NPTX2 expression levels by damaging neural networks associated with memory, such as the hippocampus and frontal lobe, leading to impaired delayed memory. As an AMPA receptor clustering factor, NPTX2 regulates glutamatergic synaptic transmission, and lower NPTX2 levels may reduce synaptic transmission efficiency, thereby affecting memory function (Schwarz et al. 2002).

This study found elevated serum NPTX2 levels in AIS patients, including both PSCI and PSNCI groups. Neuroinflammation during the acute phase of AIS triggers genetic changes, and NPTX2 may act similarly to acute‐phase proteins like C‐reactive protein in countering inflammation (Ng et al. 2018; Swanson et al. 2018). While previous studies have linked NPTX2 upregulation to neuroprotection in ischemic stroke, some suggest it may also be linked to neuronal damage (Schwarz et al. 2002; Cai et al. 2019; Thatipamula and Hossain 2014; Lu et al. 2003). In our study, NPTX2 levels in first‐episode AIS patients (PSCI and PSNCI) were higher than in the control group. However, at 3 months poststroke, levels in the PSCI group were lower than in the PSNCI group. This suggests that although AIS increases NPTX2, the rise may not be sufficient to support cognitive recovery in all patients. The lower NPTX2 levels in the PSCI group may reflect a deficiency that limits synaptic remodeling and cognitive function, leading to impairment. This underscores NPTX2's dual role: while its initial increase may be neuroprotective, insufficient levels may increase the risk of PSCI. Further research is needed to better understand these mechanisms and clarify NPTX2's role in poststroke cognitive outcomes.

However, it still had some limitations. First, although this study used a prospective design, its short‐term follow‐up limits the ability to infer causality. Long‐term follow‐ups or longitudinal designs are needed to validate the causal relationship between NPTX2 levels and long‐term cognitive changes. Second, the data were collected from a single hospital, which may limit the generalizability of the findings. Patients from other regions or hospitals might have different characteristics or treatment strategies, affecting NPTX2 levels and their association with PSCI. Multicenter studies would improve the external validity of the results. Third, serum NPTX2 levels were measured only once upon patient admission, without tracking changes over time. Since NPTX2 may behave differently during the acute and recovery phases, future research should include multiple measurements to better understand its role in PSCI development. Additionally, while some known confounding factors (e.g., age, gender, stroke severity) were controlled, unmeasured factors like lifestyle or undiagnosed conditions could still influence the relationship between NPTX2 levels and PSCI. Socioeconomic status and education, which may also affect cognitive function, were not fully considered. Finally, the MoCA scale used to assess cognitive function has limitations, particularly in detecting issues in domains like executive function and memory. Future studies should consider using additional cognitive assessment tools for a more comprehensive evaluation.

## 5 Conclusion

This study demonstrates that lower baseline serum NPTX2 levels are associated with PSCI in patients with AIS. NPTX2 could serve as a potential biomarker for identifying patients at risk of PSCI. However, further large‐scale studies are needed to confirm these results and explore the underlying mechanisms. Our results revealed that baseline serum NPTX2 levels are associated with PSCI, with lower serum NPTX2 levels considered to be a risk factor for PSCI. Our study provides further evidence for the relationship between NPTX2 levels and cognitive impairment in AIS patients, while a further larger‐scale study is needed to verify our findings.

## Author Contributions

**Jie Li**: Funding acquisition, formal analysis, supervision, investigation, validation. **Wenyang Ma**: Writing–original draft, data curation, software, methodology, project administration. **Shiyuan Gu**: Writing–review and editing, funding acquisition, conceptualization.

### Conflicts of Interest

The authors declare no conflicts of interest.

### Peer Review

The peer review history for this article is available at https://publons.com/publon/10.1002/brb3.70305.

## Data Availability

The data that support the findings of this study are available from the corresponding author upon reasonable request.
